# Regulation of vascular tone by adipocytes

**DOI:** 10.1186/1741-7015-9-25

**Published:** 2011-03-16

**Authors:** Nele Maenhaut, Johan Van de Voorde

**Affiliations:** 1 Department of Pharmacology, Ghent University, De Pintelaan 185-Blok B, 2nd floor, BE-9000 Ghent, Belgium

## Abstract

Recent studies have shown that adipose tissue is an active endocrine and paracrine organ secreting several mediators called adipokines. Adipokines include hormones, inflammatory cytokines and other proteins. In obesity, adipose tissue becomes dysfunctional, resulting in an overproduction of proinflammatory adipokines and a lower production of anti-inflammatory adipokines. The pathological accumulation of dysfunctional adipose tissue that characterizes obesity is a major risk factor for many other diseases, including type 2 diabetes, cardiovascular disease and hypertension. Multiple physiological roles have been assigned to adipokines, including the regulation of vascular tone. For example, the unidentified adipocyte-derived relaxing factor (ADRF) released from adipose tissue has been shown to relax arteries. Besides ADRF, other adipokines such as adiponectin, omentin and visfatin are vasorelaxants. On the other hand, angiotensin II and resistin are vasoconstrictors released by adipocytes. Reactive oxygen species, leptin, tumour necrosis factor α, interleukin-6 and apelin share both vasorelaxing and constricting properties. Dysregulated synthesis of the vasoactive and proinflammatory adipokines may underlie the compromised vascular reactivity in obesity and obesity-related disorders.

## Introduction

For a long time, adipose tissue or body fat was believed to be simply involved in total body lipid and overall energy homeostasis. White adipose tissue stores excess energy in the form of triglycerides, while brown adipose tissue is actively involved in the regulation of body temperature [1,2]. However, in recent years, it has become clear that adipose tissue is far more than a storage facility and thermoregulator and is in fact an active secretory organ of multiple mediators known as adipokines [3]. These adipokines include hormones (for example, leptin and adiponectin), inflammatory cytokines (for example, tumor necrosis factor α (TNFα), interleukin (IL)-6, omentin and visfatin) and other proteins (for example, plasminogen activator inhibitor (PAI)-1, angiotensinogen, resistin and apelin) [4,5]. Furthermore, adipose tissue is known to release an as yet unidentified adipocyte-derived relaxing factor (ADRF) [6] which relaxes several arteries. Here we give an overview of the influence of different adipokines on vascular tone and on their potential role in obesity and obesity-related disorders.

## Adipokines

Adipose tissue (see "Adipose tissue" text box below) is known to produce and release numerous bioactive substances, known as adipokines, into its direct surroundings (auto- or paracrine) and into the bloodstream (endocrine) [3]. Adipokines are involved in various physiological processes (Table 1), including the regulation of arterial tone [4,7]. Therefore, adipose tissue affects not only overall metabolism but also the functionality of many organs and tissues, such as muscle, liver, brain and the vasculature. Total absence of adipose tissue has been reported to be associated with nonviability, which emphasizes the essential role of adipose tissue in human physiology [8]. Maintenance of a normal amount of adipose tissue is essential because imbalance can cause serious health problems and dysregulated release of adipokines may lead to vascular disturbances and inflammation.

**Table 1:** Physiological processes in which adipokines are involved^a^

Physiological processes	Adipokines involved
Glucose metabolism	Adiponectin, resistin
Lipid metabolism	CETP, retinol-binding protein
Immunity	Adipsin
Inflammation	TNFα, IL-6
Coagulation	PAI-1
Maintaining normal reproduction	Leptin, ghrelin
Pancreatic β-cell function	IL-6, adiponectin, visfatin
Angiogenesis	Leptin, VEGF, HGF
Feeding behaviour	Leptin
Regulation vascular tone	ADRF, leptin, adiponectin

^a^CETP, cholesteryl ester transfer protein; TNFα, tumor necrosis factor α; IL-6, interleukin 6; PAI-1, plasminogen activator inhibitor 1; VEGF, vascular endothelial growth factor; HGF, hepatocyte growth factor; ADRF, adipocyte-derived relaxing factor.

## Vasoactive adipokines in physiology and obesity

**Figure 1:**
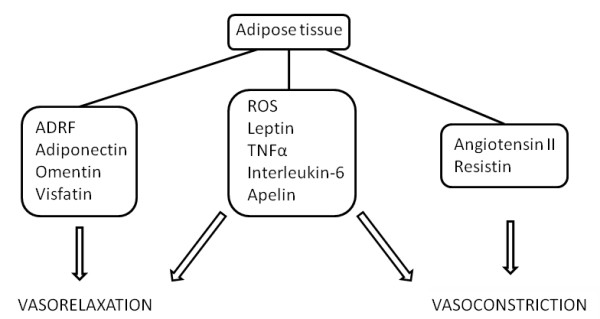
**Adipose tissue releases several adipokines**. Some of them have vasorelaxing or vasocontractile properties, while others share both. ADRF, adipocyte-derived relaxing factor; ROS, reactive oxygen species; TNFα, tumour necrosis factor α.

**Figure 2:**
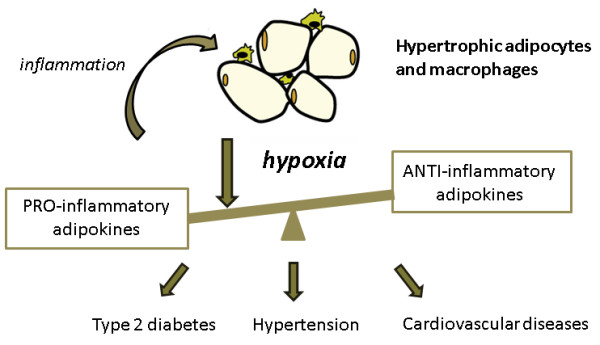
**Relationship between dysfunctional adipose tissue in obesity, inflammation, hypoxia, and obesity-related disorders**. Adipose tissue mass increases during obesity, which leads to a state in which the adipose tissue becomes hypoxic. There is a dysregulation in the synthesis of adipokines in favor of the proinflammatory ones. This might lead to obesity-related disorders and results in inflammation within adipose tissue. Hypoxia may underlie this inflammatory response by supporting the production of proinflammatory adipokines.

Under normal circumstances, vascular tone is influenced by adipokines (Figure 1 and Table 2). However, it is thought that vascular tone regulation is compromised in obesity and obesity-related disorders, in which the amount of adipose tissue has grown out of proportion. This eventually leads to a dysregulated synthesis of vasoactive adipokines by dysfunctional adipose tissue in favour of harmful proinflammatory adipokines (for example, leptin) [7] (Figure 2). The dysregulated synthesis and/or secretion of adipokines and the infiltration of macrophages into adipose tissue, possibly as a result of monocyte chemoattractant protein (MCP)-1 [9] and leptin [10] release from adipocytes, lead to a state of inflammation within adipose tissue. A proinflammatory state in adipose tissue can induce not only a dysregulation of vascular tone but also local insulin resistance, adhesion of monocytes, vascular remodelling, foam cell formation in the arterial wall and endothelial dysfunction. Endothelial dysfunction is reflected as a decrease in nitric oxide (NO) bioavailability, endothelium-dependent relaxation and impaired ability of the endothelium to respond to circulating hormones. All of these changes clearly promote the development of cardiovascular diseases and type 2 diabetes [11].

**Table 2:** Vasoactive effect of adipokines^a^

Adipokines	Vasoactive effect	References
Superoxide anion	Vasoconstriction through Ca^2+ ^sensitization; impairs EC-dependent relaxation by decreasing NO bioavailability; enhances vasoconstriction to perivascular nerve activation by electrical field stimulation	[18,19,29]
Hydrogen peroxide	EC-dependent and EC-independent vasorelaxation mediated by opening K_Ca_, K_v _and K_ATP _channels; Ca^2+^-dependent and Ca^2+^-independent vasoconstriction	[20,21,23-27]
Leptin	Vasoconstriction due to sympathetic nervous system activation; EC-dependent and EC-independent vasorelaxation	[31-34,38,39]
TNFα	EC-dependent and EC-independent vasorelaxation; triggers ET-1- and Ang-induced vasoconstriction; impairs EC-dependent vasorelaxation due to decreased NO or increased ROS production; reduces vasorelaxing effect of PVAT due to increased ROS production	[50,52,53,57-59]
IL-6	EC-independent vasorelaxation; reduces vasorelaxing effect of PVAT due to increased ROS production; impairs endothelial function due to increased ROS and decreased NO production	[59,66,67,72]
Apelin	NO-dependent vasorelaxation; EC-independent vasoconstriction	[76-78,80]
Adiponectin	NO-dependent vasorelaxation mediated by opening K_v _channels	[59,90,91]
Omentin	EC-dependent and EC-independent vasorelaxation	[100]
Visfatin	NO-dependent vasorelaxation	[107]
ADRF	Vasorelaxation through opening of K_ATP_, KCNQ or K_Ca _channels depending on the species	[6,92,112,114,115]
Ang II	Vasoconstriction via binding on AT1 receptors	[124]
Resistin	No effect on contractility of blood vessels; impairs endothelial function due to increased ET-1 production and decreased NO production	[135,144]

^a^EC, endothelial cell; NO, nitric oxide; K_Ca _channels, Ca^2+^-activated K^+ ^channels; K_v _channels, voltage-dependent K^+ ^channels; K_ATP _channels, ATP-sensitive K^+ ^channels; TNFα, tumor necrosis factor α; ET-1, endothelin 1; Ang, angiotensinogen; ROS, reactive oxygen species; PVAT, perivascular adipose tissue; IL-6, interleukin 6; Ang II, angiotensin II; AT1, angiotensin type 1; ADRF, adipocyte-derived relaxing factor.

It has been proposed that hypoxia underlies this inflammatory response, as hypoxia occurs in areas of fat depots when the vascular oxygen supply is compromised because of tissue mass expansion [4]. Direct evidence that growing adipose tissue becomes hypoxic has recently been shown in mice [12,13]. Furthermore, cell culture studies using murine and human adipocytes strongly support the modulatory role of hypoxia in the production of several proinflammatory adipokines [14,15].

Furthermore, angiogenesis is promoted in response to hypoxia [16]. Novel vascularisation can be considered an automatic fail-safe to counter hypoxia and ensure sufficient nutrient and oxygen supply to the different tissues. Hypoxia upregulates inducible transcription factors, which trigger the expression of angiogenic adipokines such as vascular endothelial growth factor (VEGF), hepatocyte growth factor and PAI-1 [7], which promote vascular endothelial cell proliferation and the later stages of new vessel formation [17]. Also, other adipokines, such as leptin, basic fibroblast growth factor and IL-6, have been shown to induce angiogenesis, while adiponectin and TNFα have pro- and antiangiogenic properties [17]. The vasoactive adipokines and their role in physiological conditions as well as in obesity and obesity-related disorders are described in more detail in the following subsection.

### Adipokines with vasorelaxing and vasocontractile properties

#### Reactive oxygen species

Reactive oxygen species (ROS) are a class of oxygen-derived molecules including superoxide anion and hydrogen peroxide, both of which are modulators of vascular tone. Vascular smooth muscle cells, endothelium and perivascular adipose tissue are known to contain ROS [18].

Superoxide anions can induce vasoconstriction through Ca^2+ ^sensitization pathways, although it is not clear whether they act directly or via conversion to hydrogen peroxide [19]. Furthermore, contraction in response to perivascular nerve activation by electrical field stimulation is enhanced by superoxide anions from perivascular adipose tissue [18].

Hydrogen peroxide is a more likely paracrine ROS because hydrogen peroxide is not a free radical and therefore more stable and less reactive with other tissue radicals [20]. Hydrogen peroxide is known to induce both vasorelaxation and vasoconstriction, depending on species, type of vascular bed, concentration, membrane potential and degree of obesity [20-22]. Vasorelaxation is possibly induced by endothelium-dependent mechanisms involving the release of vasodilating cyclooxygenase metabolites [23] and NO [24], as well as endothelium-independent mechanisms [21] mediated by the activation of different potassium channels on smooth muscle cells [23,25,26]. On the other hand, vasoconstriction by hydrogen peroxide is likely induced in a Ca^2+^-dependent way, although Ca^2+ ^sensitization and Ca^2+^-independent pathways have also been reported [20,24,27]. Furthermore, hydroxyl radicals, cyclooxygenase metabolites, protein kinase C, phospholipase A_2 _phospholipase C and tyrosine kinase appear to play a role in hydrogen peroxide-induced contractions [27].

Oxidative stress occurs when the production of ROS exceeds the cell's capacity to detoxify these potentially injurious oxidants using antioxidant defense systems [28]. In general, superoxide and hydrogen peroxide production in adipose tissue is increased in obese mice, which promotes endothelial dysfunction. Superoxide anions impair endothelium-dependent vasorelaxation by decreasing NO bioavailability via the formation of peroxynitrite, which is in turn another ROS [29]. Furthermore, ROS contributes to endothelial dysfunction by upregulating the expression of adhesion and chemotactic molecules in endothelial cells, which promote monocyte adhesion and migration to the vessel wall [28]. The adhesion of these circulating blood cells to vascular endothelium is a key element in the development of inflammation and thrombosis within the vasculature in vascular diseases associated with oxidative stress, such as atherosclerosis [28].

#### Leptin

Leptin is almost exclusively secreted by white and brown adipocytes [30]. Under normal conditions, leptin contributes to blood pressure homeostasis by its vasorelaxing and vasocontractile effects [31,32]. While the contractile effect of leptin is attributed to sympathetic nervous system activation [31], various mechanisms seem to be responsible for leptin-induced vasorelaxation. This latter effect can be endothelium-dependent, either through the release of NO [33] or by other mechanisms [32,34]. The involvement of the endothelium-derived hyperpolarizing factor (EDHF) in leptin-induced vasorelaxation remains controversial [32,35]. It has been postulated that epoxyeicosatrienoic acids (EETs) and/or EDHF-dependent vasorelaxation *in vivo *might act as a backup in case of reduced NO availability [36]. On the other hand, EETs are able to activate endothelial NO synthase and subsequently release NO to influence arterial tone [37]. There is also evidence that leptin affects vascular tone without endothelial involvement [38]. A study on endothelium-denuded rat aortic rings showed that leptin attenuated angiotensin II (Ang II)-induced contraction by inhibiting Ca^2+ ^release from the intracellular stores in vascular smooth muscle cells [39].

Leptin levels are markedly increased in obesity [22,40]. Hyperleptinemia in obesity is believed to dysregulate blood pressure, resulting in hypertension. Significant associations have been found between plasma leptin levels and hypertension in both males and females, which makes leptin a potential predictor of hypertension [41,42]. In obesity, endothelium-dependent vasorelaxation is likely to become less effective, as sustained hyperleptinemia leads to endothelial dysfunction [43]. This might be the result of a leptin-induced increase of vasoconstrictor endothelin-1 [44], a leptin-induced expression of endothelin type A receptors in vascular smooth muscle cells [45], a leptin-induced depletion of NO and an increase of cytotoxic ROS [46]. Leptin also promotes smooth muscle cell proliferation, contributing to the increased peripheral vascular resistance [47]. Furthermore, it stimulates the release of proinflammatory cytokines from macrophages, which may further elevate blood pressure and exacerbate the inflammatory process [48].

#### Tumor necrosis factor α

The cytokine TNFα is a potent, time-dependent vasoconstrictor [49,50] and vasodilator [51-54]. Besides time dependency, it is unclear what underlies the differential regulation of arterial contractility by TNFα. Vasoregulatory actions of TNFα may be vascular bed-specific. Also, differences in experimental protocols used may explain the diversity of observations reported in various studies.

A source of TNFα that has recently been identified is perivascular adipose tissue [55]. This implies that TNFα is produced in the direct vicinity of the vascular endothelium. TNFα-mediated vasoregulation can occur through both endothelium-dependent [52] and endothelium-independent mechanisms [53]. Some studies have suggested that TNFα promotes vasorelaxation by an increase of NO and prostaglandin production [52,54], while another study has suggested the involvement of hydrogen peroxide [56].

On the other hand, TNFα is able to induce vasoconstriction by increasing endothelin-1 [57] and angiotensinogen levels [58]. In addition, TNFα impairs endothelium-dependent vasorelaxation in various vascular beds as a result of a decrease in endothelial NO release or an increase in NO scavengers such as ROS [50]. Moreover, a recent study has shown a reduced vasorelaxing effect of perivascular adipose tissue in response to TNFα and IL-6, which upregulate ROS [59].

Increased adipose tissue expression of TNFα mRNA has been reported in different rodent models of obesity as well as in clinical studies involving obese patients [40]. TNFα is considered a molecule that links inflammation to obesity [40]. Moreover, the infiltration of macrophages in adipose tissue during obesity contributes to increased TNFα production [60]. The increase in TNFα expression induces the production of ROS, resulting in endothelial dysfunction in obesity and obesity-related disorders such as hypertension, atherosclerosis and type 2 diabetes [61]. Furthermore, TNFα decreases adiponectin expression [62] and stimulates the secretion of proinflammatory proteins (for example, IL-6), which contribute to the maintenance of the chronic inflammatory state of adipose tissue in obesity [63].

#### Interleukin 6

A sustained increase in proinflammatory cytokine IL-6 plasma levels is associated with high blood pressure [64,65]. On the other hand, acute exposure of IL-6 *in vitro *relaxes the aorta [66]. This vasorelaxing effect is likely regulated by an endothelium-independent pathway involving an increase in prostacyclin in vascular smooth muscle cells. IL-6 also relaxes skeletal muscle resistance vessels. However, this occurs only *in vivo*, suggesting that IL-6 interacts with parenchymal or intravascular factors to elicit vasorelaxation [67].

In obesity, an increase in cytokine IL-6 has been observed at the mRNA and protein levels in white adipose tissue [68,69]. IL-6 has been shown to be a predictor of future myocardial infarction [65] and is highly associated with cardiovascular mortality [70]. IL-6 induces the induction of hepatic C-reactive protein (CRP) production, which is now known to be an independent major risk factor for cardiovascular complications [40]. Some studies have suggested that IL-6 is rather an indirect marker of vascular dysfunction, while others have suggested a more active role in vascular dysfunction [71]. Long-term elevation of IL-6 in mice has been shown to impair endothelial function by increasing angiotensin II-stimulated production of ROS as well as by reducing endothelial NO synthase mRNA expression [72]. In addition, IL-6 enhances vascular smooth muscle cell proliferation [73], which is a key event in the genesis of atherosclerotic lesions.

Genetic deletion of IL-6 attenuates angiotensin II-induced hypertension in mice [64], suggesting that elevated IL-6 in obesity might contribute to hypertension via Ang II. In addition, IL-6 inhibits adiponectin gene expression in cultured adipocytes [68], which may exacerbate obesity-related hypertension.

#### Apelin

Apelin, of which different isoforms exist, acts through the binding to a specific G protein-coupled receptor named APJ [74], which is present on endothelial cells, vascular smooth muscle cells and cardiomyocytes [75]. Apelin causes NO-dependent vasorelaxation of human arteries both *in vitro *and *in vivo *[76,77]. *In vivo *exogenous apelin administration has been shown to cause a rapid NO-dependent fall in blood pressure in a rodent model, confirming its powerful vasorelaxing effect [78]. However, some reports have associated apelin with an increase in arterial pressure [79]. It has been proposed that apelin-induced changes in blood pressure (that is, an increase or decrease) are both dose- and time-dependent [74]. Furthermore, it is also possible that the observed bioactivity of apelin varies depending on species and/or vascular bed. Other data also suggest that apelin has vasoconstrictive potential by acting directly on vascular smooth muscle cells. In endothelium-denuded isolated human veins, apelin has been shown to be a potent vasoconstrictor with nanomolar potency and a maximum response comparable to that of Ang II [80]. In the presence of functional endothelium, this vasoconstrictive effect may be counterbalanced or even masked by activation of APJ receptors on vascular endothelial cells, resulting in the release of endothelial vasodilator substances such as NO [81]. All of these data taken together suggest a role for the apelin-APJ system as a regulator of vascular tone.

Apelin production in adipose tissue is strongly upregulated by insulin, and plasma concentrations are increased in obese and hyperinsulinemic mice and humans [82]. In contrast to acute exposure, long-term exposure of apelin does not affect blood pressure [83], which might be explained by resistance to its hypotensive effect. This is in contrast to a study in which high apelin levels were found to increase blood pressure in obesity via stimulation of sympathetic outflow in the central nervous system when crossing the blood-brain barrier [84].

In atherosclerosis, apelin might have beneficial effects, as apelin has been shown to stimulate endothelial NO production and antagonize the Ang II-induced formation of atherosclerotic lesions and aortic aneurysms in a murine model of atherosclerosis [85].

### Vasorelaxing adipokines

#### Adiponectin

Adiponectin is mainly released by both brown [86] and white [69] adipocytes and is the most abundant adipokine in the circulation [87]. Adiponectin has been considered an anti-inflammatory and antioxidative adipokine that protects against cardiovascular disease [88]. Adiponectin inhibits TNFα production and other inflammatory pathways in adipocytes and macrophages [40,88]. Plasma adiponectin has been correlated with endothelium-dependent vasorelaxation in humans [89]. These results were confirmed by other studies that have shown an increase in NO production as well as NO-mediated and potassium channel-mediated (that is, voltage-dependent) vasorelaxation in rats by adiponectin [59,90,91]. NO release from the endothelium is likely stimulated by adiponectin's binding to either the adiponectin type 2 receptor or T-cadherin on the endothelial surface [59]. Increased NO production inhibits platelet aggregation, leucocyte adhesion to endothelial cells and vascular smooth muscle cell proliferation. Furthermore, it reduces oxidative stress by decreasing ROS production in endothelial cells. All of these effects protect the vascular system against endothelial dysfunction [88].

The use of an adiponectin receptor 1-blocking peptide has been found to abolish the vasorelaxing effect of human perivascular adipose tissue [59]. However, vasorelaxation induced by perivascular adipose tissue remained unchanged in adiponectin gene-deficient mice [91]. It is possible that this vasorelaxing effect of perivascular adipose tissue in the adiponectin gene-deficient mice might be the result of an endothelium-independent pathway [92]. Despite the latter findings, adiponectin remains an important vasoactive regulator.

Many studies on obesity-related diseases (for example, type 2 diabetes and hypertension) [40,93], but not all [22,94], have reported an overall decrease in adiponectin levels. Hypoadiponectinemia causes endothelial dysfunction by increasing superoxide anion production [95] by promoting the production of adhesion molecules in endothelial cells and the proliferation of smooth muscle cells [96]. Low adiponectin levels have recently emerged as an independent predictor of early atherosclerosis in obese patients [96]. However, after the establishment of atherosclerosis, this association may become weaker, especially in the presence of conditions inducing a hypercatabolic state (such as heart or renal failure) which are associated with increased plasma adiponectin, accelerated progression of atherosclerosis and worse clinical outcome [88]. In fact, several data show that high circulating adiponectin levels are associated with increased cardiovascular mortality in patients with coronary artery disease [88]. Therefore, hypoadiponectinemia may have clinical value at the early stages of atherogenesis, but at more advanced disease stages its role as a meaningful biomarker is questionable.

Although whether low levels of adiponectin predict hypertension remains controversial [42,97] and whether adiponectin levels in hypertension are decreased [87,98], low adiponectin levels might contribute to the pathogenesis of obesity-related hypertension. Considering all the beneficial effects of adiponectin on the vascular system, an antihypertensive therapy which increases adiponectin levels could be of great value. It has already been demonstrated in obese adiponectin-knockout mice with hypertension that adiponectin replenishment lowers elevated blood pressure [99]. Existing drugs such as peroxisome proliferator-activated receptor γ agonists (thiazolidinediones), some angiotensin type 1 receptor blockers (telmisartan), angiotensin-converting enzyme inhibitors and cannabinoid type 1 receptor blockers (rimonabant and taranabant) have been shown to increase circulating adiponectin levels [88]. However, future strategies should focus on upregulation of adiponectin expression (and/or its receptors) or on targeting adiponectin receptors through the development of specific agonists.

#### Omentin

Omentin is a recently identified adipose tissue-derived cytokine consisting of 313 amino acids and is mainly expressed in visceral rather than in subcutaneous adipose tissue [100]. Omentin consists of two isoforms in which omentin-1 appears to be the major isoform in human plasma [101]. Furthermore, higher plasma omentin-1 levels were detected in women compared with men [101]. In isolated rat aorta, omentin directly induces an endothelium-dependent relaxation which is mediated by NO. Omentin is even capable of inducing vasorelaxation in an endothelium-independent way. Omentin-induced relaxation is also observed in isolated rat mesenteric arteries, indicating the effectiveness of omentin in resistance vessels [100]. Since only *in vitro *studies on isolated blood vessels have been performed, *in vivo *studies are necessary to explore the influence of omentin on blood pressure and its chronic influence on vascular reactivity.

Very little is known about this novel protein in obesity. What is known is that omentin plasma levels and adipose tissue gene expression are decreased in obesity [101] and even more when overweight is combined with type 2 diabetes [102]. Furthermore, decreased omentin-1 levels are associated with low plasma adiponectin and high-density lipoprotein levels. In addition, omentin-1 levels are negatively correlated with leptin levels, waist circumference, body mass index and insulin resistance [101]. Like adiponectin, circulating omentin-1 concentrations increase after weight loss-induced improvement of insulin sensitivity [103]. Although further research is necessary, elevating omentin levels might be of interesting therapeutic value in obesity and obesity-related disorders.

#### Visfatin

Visfatin is another novel identified cytokine which is released from perivascular and visceral adipose tissue and which has an insulin-mimetic effect [104,105]. Visfatin has multiple functions in the vasculature. It stimulates growth of vascular smooth muscle cells [106] and endothelial angiogenesis via upregulating VEGF and matrix metalloproteinases [104]. Visfatin can also directly affect vascular contractility. Visfatin has been shown to induce endothelium-dependent vasorelaxation in rat isolated aorta through NO production. Also, in mesenteric arteries of rats, visfatin induces relaxation, suggesting that visfatin is effective in resistance vessels [107]. Because only acute effects of visfatin have been demonstrated, further studies are necessary to explore the chronic influence of visfatin on vascular reactivity.

Most studies, but not all, have shown an increase in visfatin levels in obesity [105,108]. A relationship of plasma visfatin levels was seen with body mass index and percentage of body fat, but not with abdominal circumference or visceral fat estimated on the basis of computed tomography [108]. It has been reported that the expression of visfatin is high at plaque rupture sites in patients with coronary artery disease [109]. Visfatin accelerates monocyte adhesion to endothelial cells by upregulating intercellular cell adhesion molecule-1 and vascular cell adhesion molecule (VCAM)-1 in vascular endothelial cells due to ROS overproduction, suggesting a possible role for visfatin in the development of atherosclerosis [110]. Further studies are necessary to clarify the atherogenic and vasoactive effects of visfatin and its potential clinical relevance.

#### Adipocyte-derived relaxing factor

Vascular tone can also be regulated by an unknown ADRF which is released from perivascular adipose tissue. Soltis and Cassis [111] first described that the presence of perivascular adipose tissue reduced vascular contractions by norepinephrine in rat aorta, which was later confirmed by Löhn *et al*. [6]. Also, isolated adipose tissue and cultured rat adipocytes relaxed precontracted rat aorta previously cleaned of adherent adipose tissue. This modulatory effect was attributed to ADRF, which functions as a regulator of arterial tone by active antagonism of contraction [6]. A similar vasorelaxing effect of perivascular adipose tissue was observed in rat mesenteric arteries [112], in mouse aorta [113] and in human internal thoracic arteries [114]. These data suggest a common pathway for arterial tone regulation in different species and different types of vascular structures. Verlohren *et al*. [112] even showed a positive correlation between the vasorelaxing influence of ADRF and the amount of perivascular adipose tissue. The observation that the resting membrane potential of vascular smooth muscle cells in arteries with adipose tissue is more hyperpolarized than in arteries without adipose tissue, further supports the idea that perivascular adipose tissue actively contributes to basal arterial tone [112]. Whether NO formation and endothelium are involved in the vasorelaxation effect of ADRF is still a matter of debate [6,92,112]. On the other hand, the vasorelaxing effect of ADRF is likely mediated by the opening of different K^+ ^channels in vascular smooth muscle cells, depending on the tissue and species studied [6,92,112,114,115]. These divergent observations suggest a different distribution of K^+ ^channels in different vessels and/or species or the existence of different ADRFs.

More and more evidence is accumulating in support of the existence of different ADRFs. Löhn *et al*. [6] first suggested that ADRF is a protein. Furthermore, analyses of adipose tissue secretion in a recent electrophoresis study resulted in the visualization of different protein bands with different molecular masses (13.8 to 74.0 kDa), which may include ADRF [116]. A possible candidate is peptide angiotensin [1-7], which is a vasodilator located within adipose tissue surrounding rat aorta [117]. Blocking this particular peptide inhibits the vasorelaxing effect of perivascular adipose tissue surrounding rat aorta [117]. This hypothesis is contradicted, however, by the fact that certain ADRF-related potassium channels (K_ATP _or K_v_) [6,115] are not involved in this observed vasorelaxing effect. In addition to proteins, hydrogen peroxide produced from the NAD(P)H oxidase in adipocytes has been described as being involved in the endothelium-independent pathway of ADRF [92]. Also hydrogen sulphide has been proposed as a novel candidate of ADRF or at least as a mediator in the ADRF effect [115,118], which is consistent with inactivation of ADRF by heating (65°C for 10 minutes) [6]. Hydrogen sulphide has recently been described as a gasotransmitter generated by cystathionine γ-lyase (CSE) in perivascular adipose tissue [119,120]. Blocking of CSE inhibits the vasorelaxing effect of perivascular adipose tissue in rat aorta and mouse mesenteric arteries [115,118]. Moreover, hydrogen sulphide-induced vasorelaxation of rat aorta was inhibited by a particular ADRF-related potassium channel (KCNQ) blocker [115]. However, hydrogen sulphide generation and CSE expression in the perivascular adipose tissue of stenotic aortas (but not in aortic tissue) have been shown to be increased in rat hypertension induced by abdominal aortic banding [118], while the vasorelaxing effect of perivascular adipose tissue has been shown to be impaired in spontaneously hypertensive rats [121]. This might indicate that ADRFs other than hydrogen sulphide are impaired, resulting in a reduced vasorelaxing effect of adipose tissue. On the other hand, it is difficult to compare both studies, as they used different models of hypertension. Furthermore, the upregulation of CSE and hydrogen sulphide generation in perivascular adipose tissue of stenotic aortas may have developed independently of hypertension, as CSE-knockout mice have been shown to be hypertensive [120].

Obesity is characterized by a decrease in the vasorelaxing effect of perivascular adipose tissue, leading to hypertension [22,59,91,122]. This might imply a decrease in ADRF release or an imbalance in adipose tissue-derived relaxing and vasocontractile factors during obesity. On the other hand, hypoxia, which develops within adipose tissue during obesity [12], has recently been shown to enhance the release of vasorelaxing factors released from adipose tissue, which might implicate ADRF [123]. So, the release of ADRF in obesity warrants further research.

### Vasocontractile adipokines

#### Angiotensinogen and Ang II

Brown and white adipocytes are rich sources of angiotensinogen, the precursor protein of a major vasocontractile peptide called Ang II [124], and possess all the enzymes necessary to produce Ang II [125]. These findings suggest the existence of a local renin-angiotensin system in adipose tissue. Moreover, the amount of angiotensinogen mRNA in adipose tissue is 68% of that in the liver, supporting an important role for adipose angiotensinogen in Ang II production [126]. The importance of this angiotensinogen source in blood pressure regulation by the renin-angiotensin system was shown in wild-type and angiotensinogen-deficient mice in which adipocyte-derived angiotensinogen was overexpressed. When angiotensinogen expression was restricted to adipose tissue (in an angiotensinogen-deficient background), circulating angiotensinogen was detected and the mice were normotensive. On the other hand, wild-type mice were hypertensive because of the additional amount of angiotensinogen that developed as a result of overexpression of adipocyte-derived angiotensinogen [127].

An important effect of Ang II is that this peptide enhances the metabolism of NO into oxygen free radicals, which damage the vascular tissue [128]. Therefore, an imbalance between Ang II and NO leads to endothelial dysfunction, resulting in a loss of vasodilator capacity. This results in an increased expression of adhesion molecules and proinflammatory cytokines in endothelial cells, which promotes monocyte and leukocyte adhesion and migration to the vessel wall [129]. Furthermore, Ang II exerts detrimental effects on the progression and destabilization of atherosclerotic plaque because of an increased release of PAI-1, causing thrombosis and increased expression of growth factors, which leads to smooth muscle cell proliferation and migration [129]. Most data support an elevation of angiotensinogen mRNA expression in adipose tissue during obesity [130]. Furthermore, several studies have highlighted a contribution of adipose tissue-derived angiotensinogen and/or angiotensin II to obesity-related hypertension [130]. High Ang II levels may deteriorate obesity-related hypertension because of an increased secretion of proinflammatory cytokines [131], decreased adiponectin secretion [132] and increased leptin production in adipocytes [133].

#### Resistin

Resistin, which is expressed in brown and white adipose tissue, is a member of the family of cysteine-rich proteins called resistin-like molecules [86,134]. Resistin is secreted into the medium by cultured adipocytes and circulates in plasma, indicating that it is a secretory product of adipose tissue. However, circulating monocytes and macrophages in particular seem to be responsible for resistin production in humans [40]. Although resistin does not directly affect the contractility of isolated blood vessels [135], coronary blood flow, mean arterial pressure or heart rate [136], it has been associated with endothelial dysfunction and coronary artery disease [137].

Initial findings have been reported regarding an association between obesity and elevated plasma resistin levels [138,139]. However, this finding was not confirmed by other investigators [140,141]. Resistin expression is stimulated by TNFα and IL-6, both of which are increased in obesity [142], which offers an explanation for an increased level of resistin in obesity. Resistin augments endothelin-1 release, which causes endothelial dysfunction. Moreover, resistin impairs endothelial function with [143] or without [136] augmenting superoxide production, resulting in decreased expression of endothelial NO synthase and NO levels [144]. Resistin also augments the expression of VCAM-1 and MCP-1, both of which are involved in early atherosclerotic lesion formation [145]. It has also been shown that high plasma resistin levels are independently associated with an increased risk for hypertension among nondiabetic women [146].

## Conclusions

Adipose tissue produces and secretes several adipokines. Some of these adipokines possess vasoactive properties (Figure 1). Arterial tone can be controlled through the release of ROS, leptin, adiponectin, TNFα, IL-6, Ang II, omentin, resistin, visfatin, apelin and ADRF. The regulation of arterial tone might be compromised in obesity and obesity-related disorders (for example, type 2 diabetes, cardiovascular disease and hypertension) because of alterations in the secretion of vasoactive adipokines by dysfunctional adipose tissue. Circulating levels of adiponectin and omentin are decreased, while levels of leptin, resistin, apelin and proinflammatory cytokines are increased. One therapeutic strategy to counter the progression of obesity-related vascular diseases is to elevate adiponectin and omentin levels. Adiponectin levels are already elevated by the use of thiazolidinediones, telmisartan, angiotensin-converting enzyme inhibitors, rimonabant and taranabant [88]. On the other hand, the development of specific agonists to target adiponectin and omentin receptors or inhibit detrimental adipokine signalling pathways may be new and promising methods to attenuate the proinflammatory effects and ultimately to reduce the progression of obesity-related vascular diseases.

## Adipose tissue

Adipose tissue is predominantly located around blood vessels (perivascular), around internal organs (visceral or abdominal) or subcutaneously. Adipose tissue consists of a heterogeneous mixture of cellular structures (that is, adipocytes, precursor cells, macrophages, fibroblasts and endothelial cells) and tissue structures (that is, small blood vessels and nerve tissue) [147]. The predominant cell type in adipose tissue is the adipocyte, which may be white or brown. In accordance with the type of adipocytes which compose it, adipose tissue is subdivided into white and brown adipose tissue.

White adipose tissue comprises up to 20% to 25% of total body weight. In general, white adipose tissue acts mainly as an energy store or reserve (that is, lipid storage) and expands during obesity. It also provides thermal insulation (subcutaneous adipose tissue) and supports the body against mechanical shocks (for example, skin or kidney) [1].

Brown adipose tissue regulates body temperature by lipid metabolism in newborn mammals and some hibernating animals [2]. Recent studies have shown that healthy adult humans still possess a substantial fraction of metabolically active brown adipose tissue in the supraclavicular and neck regions, along with some additional paravertebral, mediastinal, paraaortic and suprarenal locations [148,149]. Although the obesity-preventive role of brown adipose tissue has long been a matter of debate, more recent data clearly show an inverse correlation between body mass index and brown adipose tissue activity in humans [148,150].

## Competing interests

The authors declare that they have no competing interests.

## Authors' contributions

NM and JVDV both meet the criteria for authorship.

## Pre-publication history

The pre-publication history for this paper can be accessed here:

http://www.biomedcentral.com/1741-7015/9/25/prepub
